# An adult with episodic retrosternal chest pain: an unusual presentation of congenital pulmonary airway malformation – case report

**DOI:** 10.1186/s13019-021-01470-6

**Published:** 2021-04-13

**Authors:** Ira Goldsmith, Joseph George, Umair Aslam, Sobaran Sharma

**Affiliations:** Department of Cardiothoracic Surgery, Swansea Bay University Health Board, Morriston Hospital, Swansea, Wales SA6 6NL UK

**Keywords:** Chest pain, Congenital pulmonary airway malformation, CPAM, Anomalous pulmonary venous drainage, APVD, VATS

## Abstract

**Background:**

Congenital pulmonary airway malformation (CPAM) with partial anomalous pulmonary venous connection presenting as episodic retrosternal chest pain on exertion in an adult has not been described.

**Case presentation:**

A 21-year-old female, non-smoker, presented with a 4-year history of sharp, episodic, retrosternal chest pains brought on with exercise. A contrast-enhanced computed tomography (CT) scan showed a grossly overinflated left lower lobe with partial anomalous pulmonary venous drainage into the left hemi-azygos vein. Lobectomy, the recommended treatment of choice, carried out thoracoscopically, was curative with satisfactory mid-term results. Histology confirmed type-II congenital pulmonary airway malformation.

**Conclusions:**

CPAM can present in young adults with unusual symptoms of chest pain on exertion. When suspected a contrast-enhanced CT scan is the gold standard for establishing the diagnosis. An anatomical lung resection is curative with satisfactory medium term results.

## Background

CPAM are multi-cystic segmental areas of lung tissue with abnormal broncho-alveolar development and hamartomatous proliferation of terminal respiratory units in a gland-like pattern (adenomatoid) without proper alveolar formation [1]. The estimated incidence is 1:1500–4000 live births [2]. Nearly all cases are diagnosed in the perinatal period and in adults CPAM are uncommon [2, 3]. CPAM appear as isolated cystic or solid intrathoracic mass lesions confined to one lobe, and based on the size of the cysts classified by Stoker et al. into three major histologic subtypes [4, 5]. Type-I CPAM are composed of variable-size cysts, with at least one dominant cyst (> 2 cm in diameter); type-II CPAM are composed of smaller, uniform cysts less than 1 cm in diameter; and type-III CPAM are a solid mass composed of microcysts [1, 4]. Like congenital lobar emphysema (CLE), CPAM cause a mass effect displacing the mediastinum to the opposite side [1, 4]. Patients with CPAM may be asymptomatic or present with symptoms, which are mainly respiratory, for example, in neonates and infants CPAM are a cause of respiratory distress, and children may present with life-threatening pulmonary infections [2, 3]. In adults CPAM are rare and a case of an adult presenting with pulmonary infection with fever, cough and chest pain previously described for type -III CPAM [2, 3, 6]. Contrast enhanced CT scan accurately delineates the location and extent of the lesion and helps identify the systemic arterial supply seen in 25% of cases and any other associated congenital anomalies [7, 8]. Surgical resection of the affected lobe via a standard thoracotomy is the recommended treatment of choice in symptomatic patients [3]. Careful histological examination of the surgically resected specimen helps confirm the diagnosis and exclude malignancy [8]. Lobectomy prevents the development of complications of pulmonary infections and the possible development of malignancy [8].

## Case presentation

**Fig. 1:**
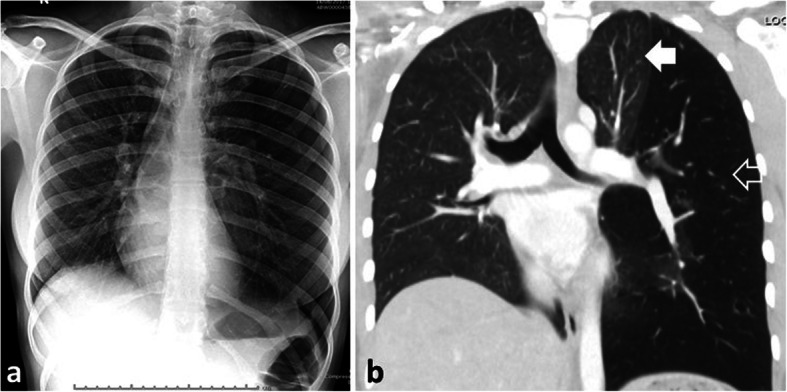
**a** Chest radiograph showing hyper-inflation of the left lung with shift of the mediastinum to the right and downward displacement of the left hemi-diaphragm. **b** Chest CT scan, coronal view, showing hyper-inflation of the left lower lobe (outlined arrow) with compression of the upper lobe (white arrow)

**Fig. 2:**
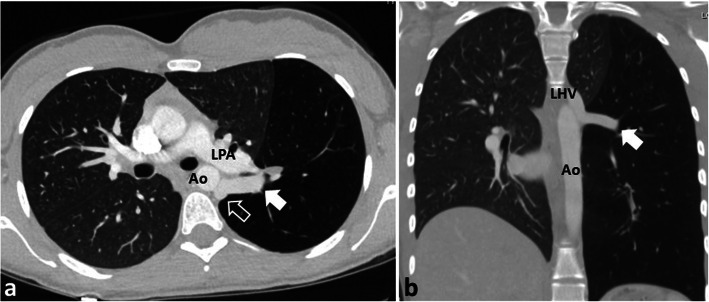
Chest CT scan, (**a**) axial view, the white arrow pointing to the inferior pulmonary vein draining into the left hemi-azygos venous system (outlined arrow), and (**b**) coronal view confirming the venous drainage into the systemic circulation. (Ao = descending aorta; LHV = left hemi-azygous vein; LPA = left pulmonary artery)

A 21-year-old female and non-smoker, presented with a 4-year history of sharp, episodic, retrosternal chest pains brought on with exercise. Exercise tolerance was however, unlimited with no breathlessness. She gave no relevant past medical history or family history of illnesses. Physical examination, ECG and an echocardiogram were normal. A chest radiograph revealed a hyperlucent left lung causing a shift of the mediastinum to the opposite side and a downward displacement of the left hemi-diaphragm (Fig. 1a). Further investigation with a contrast-enhanced CT scan confirmed the radiographic findings of a homogenous, hyperlucent, hyper-inflated left lower lobe compressing the adjacent left upper lobe and causing mediastinal and diaphragm displacement (Fig. 1b). The inferior pulmonary vein was seen draining anomalously into the left hemi-azygos vein (Fig. 2). No aberrant systemic arterial supply was present. A hyper-inflated left lower lobe suggested the diagnosis of CLE with the differential diagnosis of CPAM or pulmonary sequestration (PS).

At surgery, performed using a left video-assisted thoracoscopic (VATS) approach, the left lower lobe appeared pink, grossly enlarged, and was spongy in texture. The left inferior pulmonary vein was seen draining into the left hemi-azygos system and no aberrant systemic arterial supply was seen. The left lower lobe bronchus and pulmonary artery were normally located. A left lower lobectomy was successfully performed. The resection was challenging due to the over-distended left lower lobe despite isolating the left lung with a double lumen tube. The visceral pleural of the over-distended lobe was incised to allow any trapped air to escape. However, cutting into the spongy lung tissue failed to deflate the affected lobe and on this occasion this manoeuvre was not helpful in making the operation any easier.

On histological examination the lung parenchyma showed areas of emphysematous appearance with abnormally large air spaces, many partially lined with bronchial type epithelium and with sparse lymphoid aggregates in the alveolar walls. Respiratory bronchioles, with smooth muscle in the walls appeared unusually prominent. Within this background there were a few dilated airways showing mucous plugging, although there was no obvious evidence of bronchial atresia. There were patchy, non-specific chronic inflammation but no granuloma formation and there was no evidence of malignancy. Morphologically, the features were closest to those of a type-II CPAM.

Following surgery, the compressed left upper lobe took time to re-inflate fully. She herself made an uneventful recovery. At one-month follow-up she was pain-free, and has remained asymptomatic at 6 months and at 2 years follow-up.

## Discussion and conclusions

CPAM share similar embryologic and clinical characteristics with PS, CLE, and bronchogenic cyst (BC), the four major congenital cystic lesions that are an important cause of morbidity in neonates, infants, and children and presenting symptoms are primarily respiratory related [1, 8]. In neonates and infants these congenital lesions cause respiratory distress whilst in children and young adults the presenting symptoms are primarily respiratory distress, recurrent attacks of respiratory embarrassment, pulmonary infection, which may be associated with haemoptysis, or rarely haemothorax [2, 3, 9]. Our case report is unique where a young adult presented with symptoms of sharp, episodic, retrosternal chest pains that were brought on with exercise. This mode of presentation has not been previously described. The mechanisms that could account for the pain are unclear and can only be speculated as a result of compression of the functioning lung with displacement of the mediastinum to the opposite side during exercise (Fig. 1a), limited exercise tolerance due to less functional lung or pleuritic-type pain due to the CPAM segment. A chest radiograph is helpful in investigating the cause of this unusual mode of presentation. When suspected a contrast-enhanced CT scan is the gold standard for establishing the diagnosis and provides information about vascular anomalies such as concomitant cardiovascular, renal, musculoskeletal, gastrointestinal and other malformations [1, 9–11].

It is possible that instead of CPAM we have reported a case of PS or CLE. In our case the radiological findings of a left lower lobe involvement with its intact visceral pleura raised the possibility of PS, where the arterial blood supply arises from the systemic arteries, usually the thoracic or abdominal aorta, and its venous drainage may be via the azygous system instead of the left atrium [11]. In our case an aberrant arterial supply was not seen although there was an aberrant venous drainage into the hemiazygos system (Fig. 2). However, radiological features of an overinflated lobe causing a mass effect, a normal connection with the bronchial tree, a normal connection with the pulmonary artery, and absence of an aberrant arterial supply favoured CLE or CPAM [9, 12]. At surgery the visceral pleura of the emphysematous appearing lobe was incised to allow air to egress out and deflate the involved lobe. However, on cutting into the spongy tissue of the involved lobe there was only a very little amount of air which escaped and the lobe failed to deflate. This finding favoured CPAM instead of CLE. The diagnosis of CPAM was supported by the histological findings of the resected specimen where air spaces lined with bronchial epithelium and unusually prominent respiratory bronchioles with smooth muscle in the walls were seen. These features were those of a type-II CPAM [4].

In symptomatic patients with CPAM a surgical lobectomy via a standard thoracotomy is the recommended treatment of choice. In asymptomatic patients a prophylactic lobectomy to prevent the development of future respiratory infections and the development of malignancy is, however, debateable [2, 3]. Nevertheless, as demonstrated in our patient, when indicated a VATS approach instead of a standard thoracotomy is feasible with satisfactory medium term results.

## Data Availability

All information pertaining to the study, namely pictures, patient consent and operation notes are available for review.

## Abbreviations

Ao: Descending aorta
ECG: Electrocardiogram
LHV: Left hemi-azygous vein
LPA: Left pulmonary artery
CLE: Congenital lobar emphysema
CPAM: Congenital pulmonary airway malformation
CT scan: Computed tomography scan
PS: Pulmonary sequestration
